# Phenolic Glycosides from *Capsella* *bursa-pastoris* (L.) Medik and Their Anti-Inflammatory Activity

**DOI:** 10.3390/molecules22061023

**Published:** 2017-06-20

**Authors:** Joon Min Cha, Won Se Suh, Tae Hyun Lee, Lalita Subedi, Sun Yeou Kim, Kang Ro Lee

**Affiliations:** 1 Natural Products Laboratory, School of Pharmacy, Sungkyunkwan University, Suwon 16419, Korea; zoomin89@skku.edu (J.M.C.); angelwonse@skku.edu (W.S.S.); thlee16@skku.edu (T.H.L.); 2 Gachon Institute of Pharmaceutical Science, Gachon University, 191 Hambakmoero, Yeonsu-gu, Incheon 21936, Korea; subedilali@gmail.com (L.S.); sunnykim@gachon.ac.kr (S.Y.K.); 3 College of Pharmacy, Gachon University, 191 Hambakmoero, Yeonsu-gu, Incheon 21936, Korea

**Keywords:** *Capsella bursa-pastoris*, Cruciferae, sesquilignan glycoside, anti-inflammatory

## Abstract

A new sesquilignan glycoside **1**, together with seven known phenolic glycosides **2**–**8** were isolated from the aerial parts of *Capsella bursa-pastoris.* The chemical structure of the new compound **1** was elucidated by extensive nuclear magnetic resonance (NMR) data (^1^H- and ^13^C-NMR, ^1^H-^1^H correlation spectroscopy (^1^H-^1^H COSY), heteronuclear single-quantum correlation (HSQC), heteronuclear multiple bond correlation (HMBC), and nuclear overhauser effect spectroscopy (NOESY)) and HR-FABMS analysis. The anti-inflammatory effects of **1**–**8** were evaluated in lipopolysaccharide (LPS)-stimulated murine microglia BV-2 cells. Compounds **4** and **7** exhibited moderate inhibitory effects on nitric oxide production in LPS-activated BV-2 cells, with IC_50_ values of 17.80 and 27.91 µM, respectively.

## 1. Introduction

**Figure 1:**
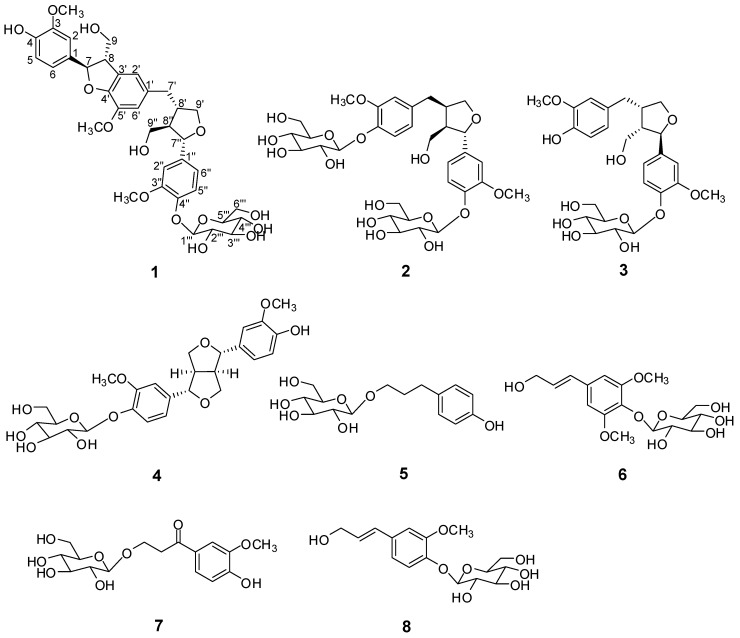
Chemical structures of compounds **1**–**8**.

*Capsella bursa-pastoris* (L.) Medik (Cruciferae), commonly known as ‘shepherd’s purse’ is an annual plant which is widely distributed throughout the world [1]. The roots of this plant have been used in Korean traditional medicine for the treatment of edema and hypertension [2]. Previous phytochemical investigations of this plant led to reports of several flavonoids, terpenoids, and phenolic compounds with antimicrobial, antibacterial, antitumor, and liver catalase activities [3,4,5,6,7,8]. Some of these compounds of *C. bursa-pastoris*, such as flavonoids and sulforaphane, have been reported to exhibit good antibacterial and anti-inflammatory activity [4,9]. In the course of our continuing search for bioactive constituents in Korean medicinal plants, we found that the *n*-BuOH fraction of the MeOH extract of *C. bursa-pastoris* inhibited NO production in a lipopolysaccharide (LPS)-activated microglia BV-2 cell line. The *n*-BuOH fraction was subjected to repeated column chromatography with Diaion HP-20, silica gel, and semipreparative HPLC separation to yield a new sesquilignan glycoside **1**, and seven known compounds **2**–**8** (Figure 1). The structure of the new compound **1** was determined by spectroscopic methods, including 1D and 2D NMR (^1^H- and ^13^C-NMR, ^1^H-^1^H COSY, HSQC, HMBC, and NOESY), and HR-FABMS analysis. The known compounds **2**–**8** were identified by comparison of their spectroscopic data and specific optical rotation with reported data. The isolated compounds **1**–**8** were tested for their inhibitory effects on NO production in an LPS-activated microglia BV-2 cell line.

## 2. Results and Discussion

### 2.1. Structure Elucidation

Purification of the *n*-BuOH layer by column chromatography afforded a new sesquilignan glycoside **1**, together with three known lignin glycosides **2**–**4** and four known phenolic glycosides **5**–**8**. The known compounds were identified as 7*S*, 8*R*, 8′*R*-(−)-lariciresinol-4,4′-bis-*O*-glucopyranoside (**2**) [10], lariciresinol4′-*O*-β-d-glucoside (**3**) [11], (+)-pinoresinol-β-d-glucoside (**4**) [12], salidroside (**5**) [13], 3-(4-β-d-glucopyranosyloxy-3,5-dimethoxy)-phenyl-2*E*-propanol (**6**) [14], β-hydroxy-propiovanillone 3*-O*-β-d-glucopyranoside (**7**) [15], and coniferin (**8**) [16] by comparing their ^1^H-,^13^C-NMR and MS spectral data with values in the literature.

Compound **1** was obtained as a brownish gum. The molecular formula was established as C_36_H_44_O_14_ using HR-FABMS, which showed a positive ion [M + Na]^+^ at *m*/*z*: 723.2626 (calcd. for C_36_H_44_O_14_Na, 723.2629, so the molecular formula was deduced to be C_36_H_44_O_14_. The ^1^H-NMR spectrum of **1** revealed the presence of two 1,3,4-trisubstituted aromatic rings (δ_H_ 7.16 (d, *J* = 8.3 Hz, H-5′′), 7.15 (d, *J* = 8.2 Hz, H-5), 7.05 (d, *J* = 1.7 Hz, H-2), 7.01 (d, *J* = 1.7 Hz, H-2′′), 6.95 (dd, *J* = 8.2, 1.7 Hz, H-6), and 6.90 (dd, *J* = 8.2, 1.7 Hz, H-6″)), a 1,3,4,5-tetrasubstitued aromatic ring (δ_H_ 6.76 (s, H-6′), 6.78 (s, H-2′)), two oxygenated methines (δ_H_ 5.58 (d, *J* = 5.9 Hz, H-7) and 4.85 (m, H-7′′)), two oxygenated methylenes (δ_H_ 3.85 (m, H-9b), 3.78 (m, H-9a), and 3.88 (m, H-9′′a), 3.68 (dd, *J* = 10.9, 6.7 Hz, H-9b′′)), a methylene 2.96 ((dd, *J* = 13.5, 5.0 Hz, H-7′a), and 2.57 (dd, *J* = 13.3, 11.0 Hz, H-7′b)), three methines (δ_H_ 3.48 (m, H-8), 2.75 (sep, *J* = 6.5, 5.5 Hz, H-8′), and 2.38 (quin, *J* = 6.9 Hz, H-8″)), three methoxy groups (δ_H_ 3.88 (s, 3-OCH_3_), 3.87 (s, 5′-OCH_3_) and 3.84 (s, 3′′-OCH_3_)), and a glucopyanosyl unit (δ_H_ 4.91 (d, *J* = 7.3 Hz, H-1′′′), 3.69 (m, H-6′′′), 3.51 (m, H-2′′′), 3.41 (overlap, H-4′′′, 5′′′) and 3.39 (m, H-3′′′). The ^13^C-NMR spectrum displayed 36 carbon signals, including 18 aromatic carbons δ_C_ 151.1 (C-3″), 147.9 (C-4′, 3′), 147.8 (C-3), 147.5 (C-4, 4″), 145.5 (C-5′), 139.7 (C-1″), 135.7 (C-1), 134.9 (C-1′), 119.7 (C-6″), 119.5 (C-6), 118.4 (C-6′), 118.2 (C-5″), 118.1 (C-5), 114.7 (C-2′), 111.5 (C-2″) and 111.3 (C-2), three oxygenated carbons δ_C_ 88.6 (C-7), 83.9 (C-7″), and 73.5 (C-9′), three methylene carbons δ_C_ 65.1 (C-9), 60.5 (C-9″), 33.9 (C-7′) and three methine carbons δ_C_ 55.7 (C-8), 54.2 (C-8″), 44.0 (C-8′), and, three methoxy carbons (δ_C_ 56.9 and 56.8 (×2)), and glucose carbons δ_c_ 103.1 (C-1′′′), 78.5 (C-3′′′), 78.4 (C-5′′′), 75.1 (C-2′′′), 71.6 (C-4′′′), and 62.6 (C-6′′′) (Table 1). 

**Figure 2:**
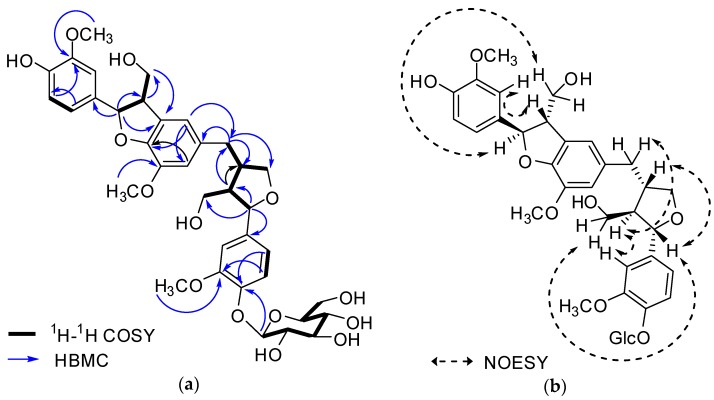
Key ^1^H-^1^H COSY, HMBC (**a**) and NOESY (**b**) correlations of **1**.

The ^1^H- and ^13^C-NMR spectra (Table 1) were very similar to those of abiesol A [17], except for the presence of a glucose unit in **1**. The positions of three methoxy groups were confirmed as 3-OCH_3_ (δ_H_ 3.88)/C-3 (δ_C_ 147.8), 5′-OCH_3_ (δ_C_ 3.87)/C-5′ (δ_C_ 145.5), and 3″-OCH_3_ (δ_H_ 3.84)/C-3″ (δ_C_ 151.1). HMBC correlation (H-1′′′ to C-4″) indicated that a D-glucose moiety was linked to C-4″ (Figure 2a), and identified the β form by the coupling constant (*J* = 7.3 Hz) [18]. The stereochemistry of **1** was assigned on the basis of examination of the CD spectrum in combination with the NOESY experiment. The absolute configurations of C-7 and C-8 were confirmed as 7*R* and 8*S* from the positive Cotton effect at 223 nm and the negative effect at 245 and 291 nm in the CD spectrum [19]. The absolute configurations of C-8′/C-7″/C-8″ were identified as 8′*S*, 7″*S* and 8*R* from the negative Cotton effect at 231 nm and 280 nm, respectively (Figure S8, Supplementary Materials) [19]. HMBC correlations and NOESY cross-peaks (Figure 2) reconfirmed the suggested structure of **1**. The enzymatic hydrolysis of **1** afforded d-glucose, which was identified by the sign of the specific rotation ${\left[\alpha \right]}_{D}^{25}$ +48.2 (*c* = 0.03, H_2_O) and by co-TLC (CHCl_3_:MeOH:H_2_O = 2:1:0.1; *R*_f_ = 0.21) [20] and the aglycone **1a**, which was identified by ^1^H-NMR and MS data [17]. Thus, the structure of **1** was determined as (7*R*, 8*S*, 8′*S*, 7″*S*, 8″*R*)-abiesol A 4″-*O*-β-d-glucopyranoside, and it was named capselloside.

### 2.2. Anti-Inflammatory Activity

Nitric oxide (NO) is one of the pro-inflammatory mediators and also a signaling molecule responsible for the induction of inflammation and pain through the pronounced induction of pro-inflammatory cytokines [21]. It is believed to be produced excessively in the inflammatory conditions so it has been considered as a major biomarker for the screening of the anti-inflammatory activity of compound [22]. Compounds that can inhibit the NO production possess an anti-inflammatory activity against LPS-induced neuroinflammation [23].

The anti-inflammatory activities of the isolates **1**–**8** were evaluated by measuring the levels of nitric oxide (NO) produced in lipopolysaccharide (LPS)-activated microglia BV-2 cells [24]. Compound **4** significantly inhibited LPS-stimulated NO production, with an IC_50_ value of 17.80 μM and compound **7** showed moderate NO production, with an IC_50_ value of 27.91 μM without cell toxicity (Table 2), which represented more potent activity than the positive control, L-NMMA (20.76 μM).

## 3. Materials and Methods

### 3.1. General Experimental Procedures

Optical rotations were measured on a P-1020 polarimeter in MeOH (Jasco, Easton, MD, USA). Infrared (IR) spectra were recorded on a IFS-66/S FT-IR spectrometer (Bruker, Karlsruhe, Germany). Circular dichroism (CD) spectra were measured on a Jasco J-715 spectropolarimeter using methanol as a solvent. Ultraviolet (UV) spectra were recorded using a UV-1601 UV-Visible spectrophotometer (Shimadzu, Tokyo, Japan). HR-FABMS spectra were obtained on a JMS700 mass spectrometer (JEOL, Peabody, MA, USA). TLC was performed using precoated silica gel F254 plates and RP-18 F254s plates (Merck, Darmstadt, Germany). NMR spectra, including COSY, HMQC, and HMBC experiments were recorded on an AVANCE III 700 NMR spectrometer at 700 MHz (^1^H) and 175 MHz (^13^C). (Bruker, Billerica, MA, USA). Preparative high performance liquid chromatography (HPLC) was conducted using a 306 pump (Gilson, Middleton, WI, USA) equipped with a Shodex refractive index detector (Shodex, New York, NY, USA) and a Phenomenex-Luna-10u-silica-(2) column (250 mm × 10.00 mm i.d.) or a YMC J’sphere ODS-M80 column (250 mm × 10.00 mm i.d.). Low-pressure liquid chromatography (LPLC) was carried out over a Li-Chroprep Lobar-A RP-18 column (240 mm × 10 mm i.d.; Merck) with an FMI QSY-0 pump (Teledyne Isco, Lincoln, NE, USA). Silica gel 60 and RP-C_18_ silica gel (230–400 mesh, Merck) were used for column chromatography. The packing material for macroporous adsorbent column chromatography was a Diaion HP-20 column (Sigma, St. Louis, MO, USA). Spots were detected by thin layer chromatography (TLC) under UV light or by heating after spraying with anisaldehyde-sulfuric acid.

### 3.2. Plant Materials

Whole plants of *C. bursa-pastoris* (3 kg) were purchased from Anmyeon-Island, Chungcheongnam-do, Korea in March 2015. A voucher specimen (SKKU-NPL 1410) has been deposited in the herbarium of the School of Pharmacy, Sungkyunkwan University, Suwon, Korea.

### 3.3. Extraction and Isolation

Whole plants of *C. bursa-pastoris* (3 kg) were extracted three times at room temperature with 80% aqueous MeOH (10 L × 1 day) and then filtered. The filtrate was evaporated under vacuum to afford a crude MeOH extract (280 g), which was suspended in distilled H_2_O and successively partitioned with *n*-hexane, CHCl*_3_*, EtOAc, and *n*-BuOH, yielding 33, 5, 4, and 26 g respectively. The BuOH-soluble layer (26 g) was separated on a Diaion HP-20 column eluted with a gradient solvent system of 100% H_2_O and 100% MeOH, yielding subfractions A (100% H_2_O) and B (100% MeOH). Subfraction B (7 g) was separated over a silica gel column (230–400 mesh, 300 g) with CHCl_3_–MeOH–H_2_O (5:1:0.1) to afford six subfractions (B1–B6). Subfraction B2 (168 mg) was chromatographed over a Lobar-A RP-18 column (25% MeCN) to give four subfractions (B21–B24). Subfraction B22 (54 mg) was purified by semi-preparative reversed-phase HPLC using a solvent system of 32% MeOH (flow rate; 2 mL/min) to isolate compound **4** (7 mg). Subfraction B24 (41 mg) was purified by semi-preparative reversed-phase HPLC using a solvent system of 45% MeOH (flow rate; 2 mL/min) to isolate compound **3** (6 mg). Subfraction B3 (1 g) was separated over a RP-C_18_ column (230–400 mesh, 150 g) with a solvent system of 10% MeOH to obtain eleven subfractions (B301–B311). Compounds **5** (7 mg), **8** (30 mg), **6** (9 mg), **7** (5 mg) and, **9** (13 mg) were acquired by purification of fractions B303 (17 mg), B304 (66 mg), B305 (21 mg), B306 (14 mg), and B311 (16 mg), respectively, using semi-preparative normal-phase HPLC with CHCl_3_–MeOH–H_2_O (5:1:0.1; flow rate; 2 mL/min). Subfraction B5 (1 g) was separated over a RP-C_18_ column (230–400 mesh, 150 g) with a solvent system of 10% MeOH to obtain ten subfractions (B501–B510). Compounds **1** (3 mg), and **2** (14 mg) were acquired by purification of fractions B503 (17 mg) and B505 (21 mg), respectively, using semi-preparative normal-phase HPLC with a solvent system of CHCl_3_–MeOH–H_2_O (2:1:0.1; flow rate; 2 mL/min).

### 3.4. Characterization

Capselloside (**1**)*:* Brownish gum; ${\left[\alpha \right]}_{D}^{25}$ −34.0 (*c* = 0.05, MeOH); UV (MeOH) λ_max_ (log ε) 280 (1.4), 228 (3.3), 214 (3.1) nm; IR (KBr) ν_max_ 3261, 2946, 2816, 1638, 1489, 1261 cm^−1^; CD (MeOH) λ_max_ (Δ ε) 291 (−), 280 (−) 257 (−) 230 (−) 223 (+); ^1^H (700 MHz) and ^13^C (175 MHz) NMR data, see Table 1; HR-FABMS (positive-ion mode) *m*/*z*: 723.2626 [M + Na]^+^ (calcd. for C_36_H_44_O_14_Na, 723.2629).

### 3.5. Enzymatic Hydrolysis

Compound **1** (1.0 mg) in H_2_O (3.0 mL) was hydrolyzed with crude β-glucosidase (10.0 mg, from almonds; Sigma-Aldrich) at 37 °C for 48 h. The reaction mixture was extracted with CHCl_3_ (3 × 3 mL). The CHCl_3_ layer (0.6 mg) was chromatographed over a silica gel Waters Sep-Pak Vac 12 cc eluted with CHCl_3_–MeOH (30:1) to give the aglycone **1a** (0.4 mg), which was identified by ^1^H‑NMR and MS data. The sugar (d-glucose) from the aqueous phase of the hydrolysate of **1** was identified by silica gel TLC (CHCl_3_–MeOH–H_2_O 2:1:0.1; *R*_f_ = 0.21) comparison with a d-glucose standard. The specific rotation of the sugar obtained from **1** was ${\left[\alpha \right]}_{D}^{25}$ +48.2 (*c* = 0.03, H_2_O).

### 3.6. Measurement of Nitric Oxide Production and Cell Viability in LPS-Activated BV-2 Cells

The inhibitory effect of the test compounds on LPS-stimulated NO production was studied using BV-2 cells. BV-2 cells were seeded on a 96-well plate (4 × 104 cells/well) and treated with or without different concentrations of the compounds. The cells were stimulated with LPS (100 µg/mL) and incubated for 24 h. The concentration of nitrite (NO_2_), a soluble oxidation product of NO, in the culture medium was measured using Griess reagent (0.1% *N*-1-naphthylethylenediamine dihydrochloride and 1% sulfanilamide in 5% phosphoric acid). Fifty microliters of supernatant was mixed with an equal volume of Griess reagent. Absorbance was measured after 10min using a microplate reader (Emax, Molecular Devices, Sunnyvale, CA, USA) at a wavelength of 570 nm. L-NMMA, a well-known nitric oxide synthase (NOS) inhibitor, was used as a positive control. Graded sodium nitrite solution was used as a standard to calculate nitrite concentration. Cell viability was evaluated by observing the ability of viable cells to reduce the yellow-colored MTT to purple formazan, using an MTT assay.

## 4. Conclusions

We have isolated and identified from *C. bursa-pastoris* a new sesquilignan glycoside **1**, which we have named capselloside, together with seven known compounds **2**–**8** and evaluated their anti-inflammatory effects. Nitric oxide synthesis inhibition potency of phenolic glycosides suggests their anti-inflammatory activity against LPS-induced neuroinflammatory model in vitro. (+)-Pinoresinol-β-d-glucoside (**4**) showed the most potent anti-inflammatory effects and β-hydroxypropiovanillone 3-*O*-β-d-glucopyranoside (**7**) showed moderate NO production in an LPS-activated microglia BV-2 cell line. Compound **4** showed better potency than that of the commercial NOS inhibitor to inhibit LPS-induced NO production and neuroinflammation. In conclusion, our results collectively support the notion that phenolic glycosides from *Capsella bursa-pastoris* (L.) Medik may be potentially responsible for the observed in vitro anti-inflammatory activity in a LPS-induced neuroinflammatory model.

## Figures and Tables

**Table 1:** ^1^H (700 MHz) and ^13^C (175 MHz) NMR data of **1** in methanol-*d*_4_ (δ in ppm) ^a^.

Position	δ_C_, Type	δ_H_ (*J* in Hz)
1	135.7, C	
2	111.3, CH	7.05, d (1.7)
3	147.8, C	
4	147.5, C	
5	118.1, CH	7.15, d (8.2)
6	119.5, CH	6.95, dd (8.2, 1.7)
7	88.6, CH	5.58, d (5.91)
8	55.7, CH	3.48, m
9	65.1, CH_2_	
a		3.78, m
b		3.85, m
1′	134.9, C	
2′	114.7, CH	6.78, s
3′	147.9, C	
4′	147.9, C	
5′	145.5, C	
6′	118.4, CH	6.76, s
7′	33.9, CH_2_	
a		2.96, dd (13.5, 5.0)
b		2.57, dd (13.3, 11.0)
8′	44.0, CH	2.75, sep (6.5, 5.5)
9′	73.5, CH_2_	
a		3.78, m
b		4.05, dd (8.3, 6.6)
1″	139.7, C	
2″	111.5, CH	7.01, d (1.7)
3″	151.1, C	
4″	147.5, C	
5″	118.2, CH	7.16, d (8.3)
6″	119.7, CH	6.90, dd (8.2, 1.7)
7″	83.9, CH	4.85, overlap
8″	54.2, CH	2.38, quin (6.9)
9″	60.5, CH_2_	
a		3.88, m
b		3.68, dd (10.9, 6.7)
Glc-1′′′	103.1, CH	4.91, d (7.3)
2′′′	75.1, CH	3.51, m
3′′′	78.5, CH	3.39, m
4′′′	71.6, CH	3.41, m
5′′′	78.4, CH	3.41, m
6′′′	62.6, CH	3.69, overlap
OCH_3_ (3″)	56.9	3.84, s
OCH_3_ (3)	56.8	3.88, s
OCH_3_ (5′)	56.8	3.87, s

^a^ The assignments were based on HSQC and HMBC experiments.

**Table 2:** Effects of compounds **1**–**8** on NO production in LPS-activated BV-2 cells.

Compound	IC_50_^a^ (µM)	Cell Viability ^b^ (%)
**1**	75.13	103.04 ± 7.08
**2**	48.80	116.68 ± 3.69
**3**	30.70	109.17 ± 8.61
**4**	17.80	117.36 ± 10.23
**5**	31.14	116.09 ± 10.67
**6**	62.21	110.02 ± 9.52
**7**	27.91	119.20 ± 6.23
**8**	49.21	111.55 ± 10.40
^c^ L-NMMA	20.76	112.89 ± 4.90

^a^ The IC_50_ value of each compound was defined as the concentration (µM) that caused 50% inhibition of NO production in LPS-activated BV-2 cells; ^b^ Cell viability after treatment with 20 µM of each compound was determined by the MTT assay and is expressed as a percentage (%). The results are averages of three independent experiments, and the data are expressed as mean ± SD; ^c^ L-NMMA was used as a positive control.

## Supplementary Materials

The NMR, HR-FABMS, IR, UV, and CD spectra of **1** are available as supplementary materials available online.
