# Supplementary material for: Phenolic Glycosides from Capsella bursa-pastoris (L.) Medik and Their Anti-Inflammatory Activity
Source: Molecules. 2017 Jun 20;22(6):1023. doi: 10.3390/molecules22061023 (PMC6152647; doi:10.3390/molecules22061023)
Supplement: Supplementary file 1 [file molecules-22-01023-s001.pdf]

Supplementary content

Phenolic glycosides from *Capsella bursa-pastoris* (L.) Medik and their biological activity

Joon Min Cha [1], Won Se Suh [1], Tae Hyun Lee [1], Lalita Subedi [2,3], Sun Yeou Kim [2,3], and Kang Ro Lee [1,*]

[1]Natural Products Laboratory, School of Pharmacy, Sungkyunkwan University, Suwon 16419, Republic of Korea

[2]Gachon Institute of Pharmaceutical Science, Gachon University, 191 Hambakmoero, Yeonsu-gu, Incheon 21936, Republic of Korea

[3]College of Pharmacy, Gachon University, 191 Hambakmoero, Yeonsu-gu, Incheon 21936, Republic of Korea

Correspondence to:

Prof. Dr. Kang Ro Lee - Tel: 82-31-290-7710; Fax: 82-31-290-7730; E-mail: krlee@skku.edu

Supporting Information Contents

Fig. **S1** ^{1}H NMR spectrum of **1** (700 MHz, CD_3OD) …………………………………………………………………………………………S5

Fig. **S2** ^{13}C NMR spectrum of **1** (175 MHz, CD_3OD) ……………………………………………………………………………………S6

Fig. **S3** HSQC spectrum of **1** ……………………………………………………………………………………………………S7

Fig. **S4** HMBC spectrum of **1** …………………………………………………………………………………………………S8

Fig. **S5** ^{1}H-^{1}H COSY spectrum of **1** ……………………………………………………………………………………………S9

Fig. **S6** NOESY spectrum of **1** …………………………………………………………………………………………………S10

Fig. **S7** HR-FAB-MS spectrum of **1** ……………………………………………………………………………………………S11

Fig. **S8** CD spectra of compound **1** ……………………………………………………………………………………………**S12**

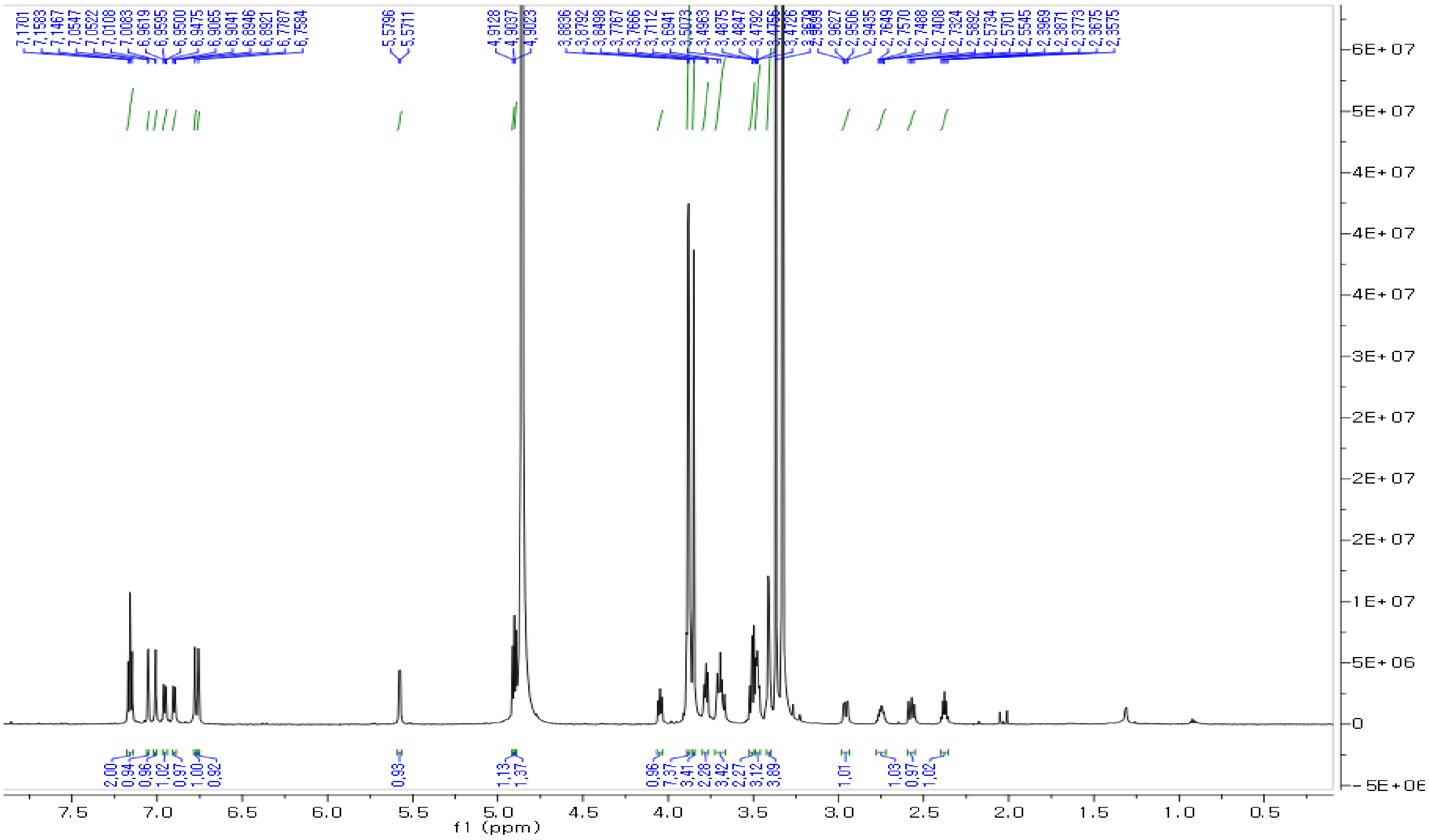

Fig. S1. ^{1}H NMR spectrum of **1** (700 MHz, CD_3OD)

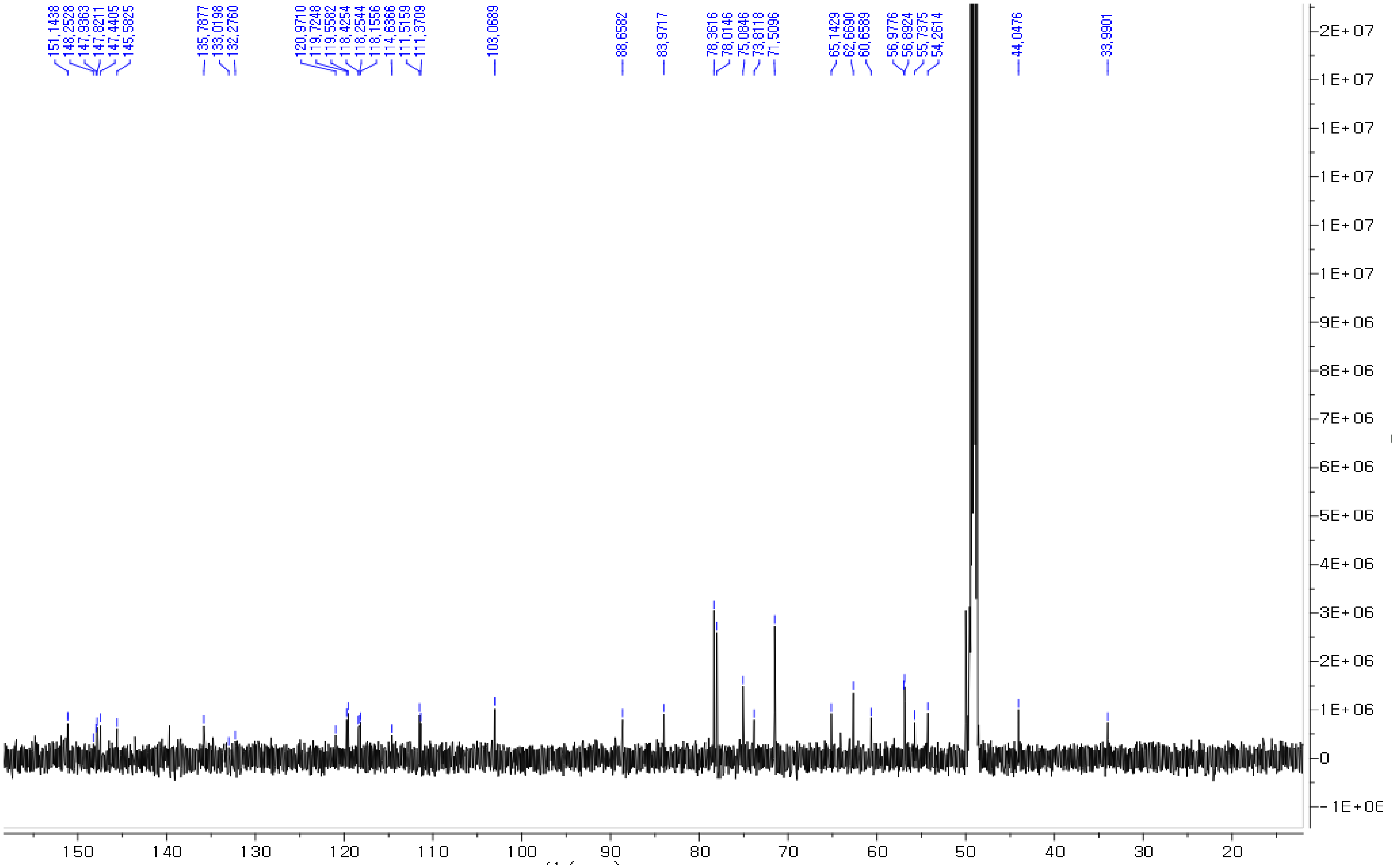

Fig. S2. ^{13}C NMR spectrum of **1** (175 MHz, CD_3OD)

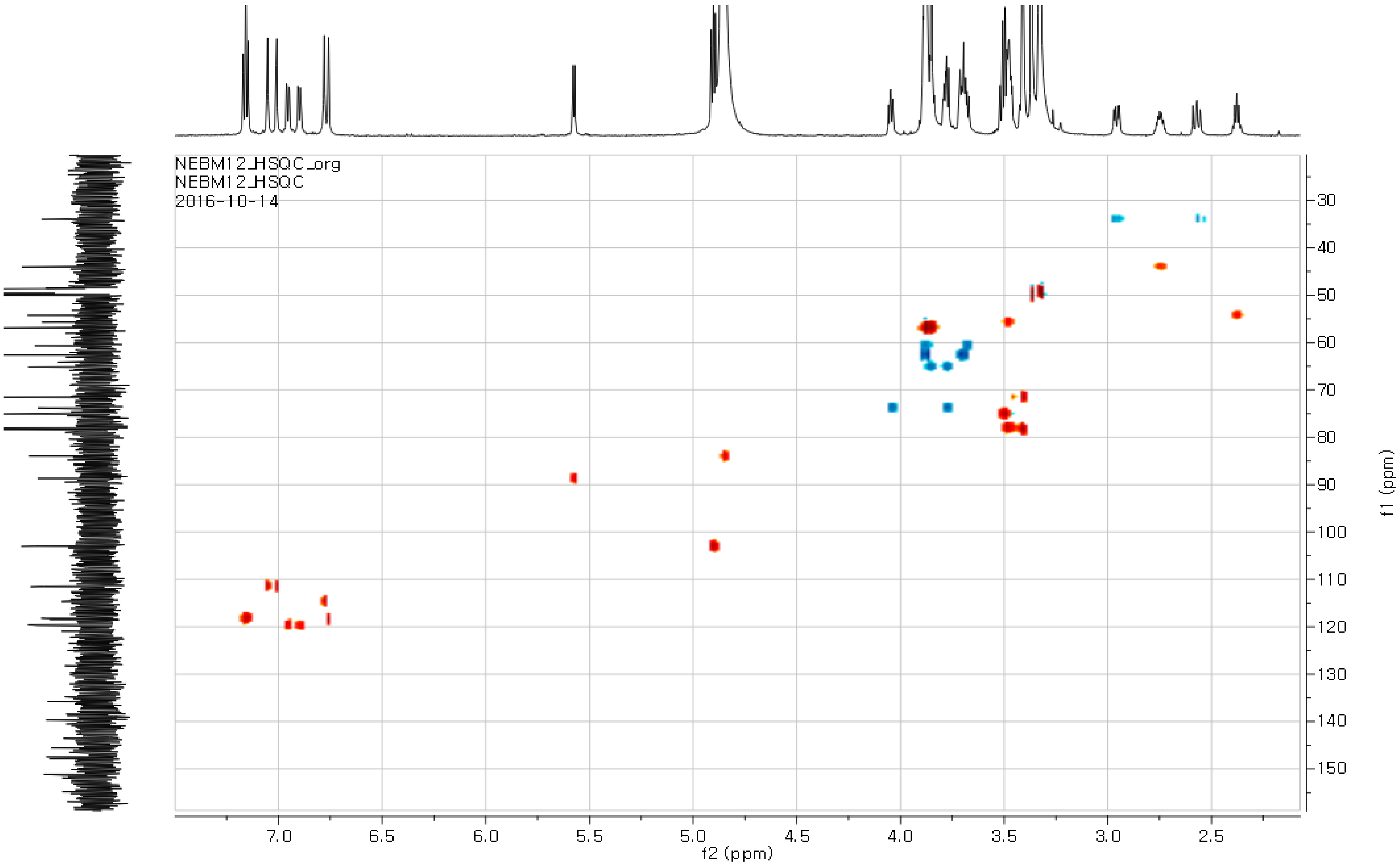

Fig. S3. HSQC spectrum of **1**

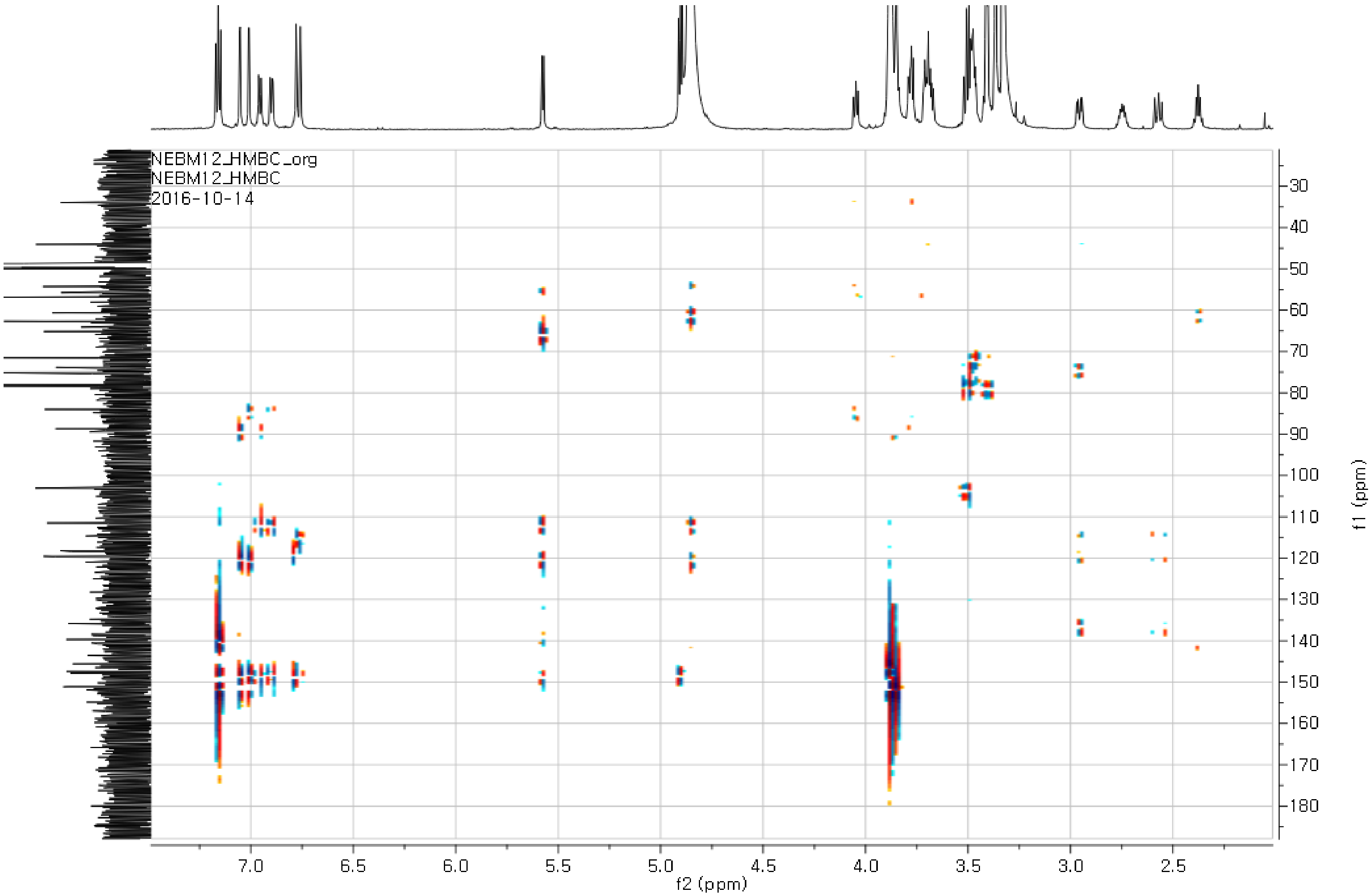

Fig. S4. HMBC spectrum of **1**

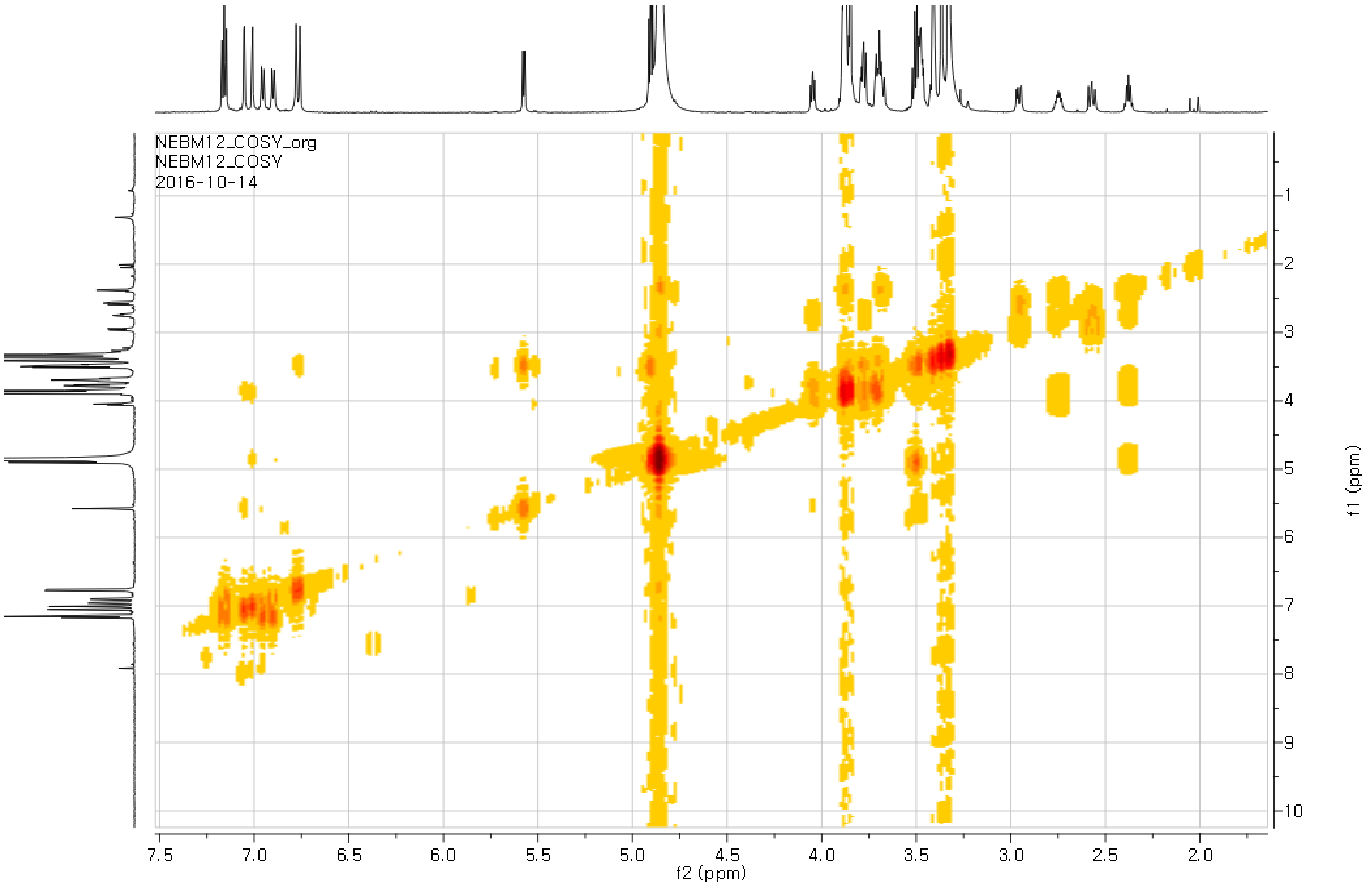

Fig. S5. NOESY spectrum of **1**

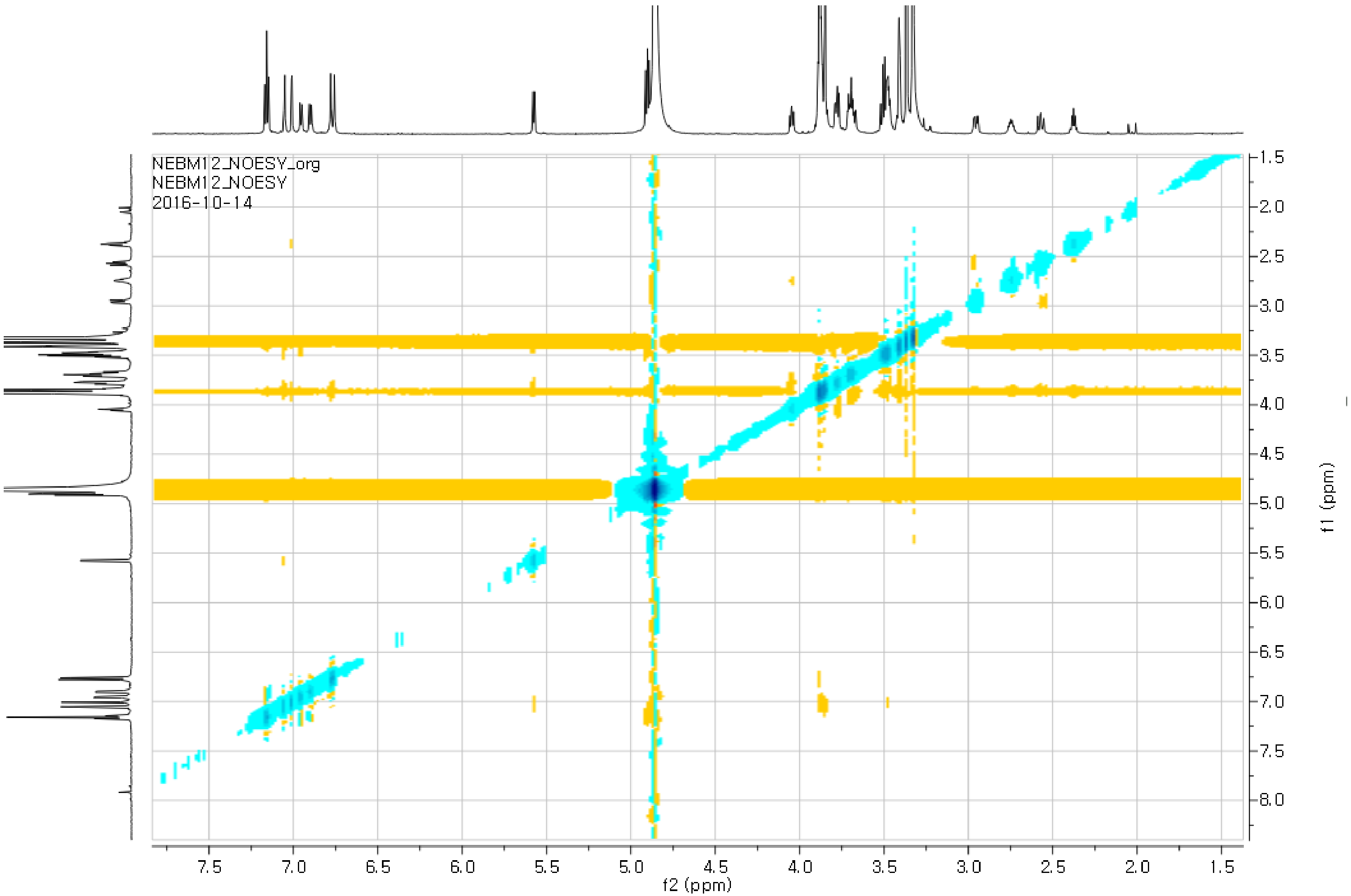

Fig. S6. ^{1}H-^{1}H COSY spectrum of compound **1**

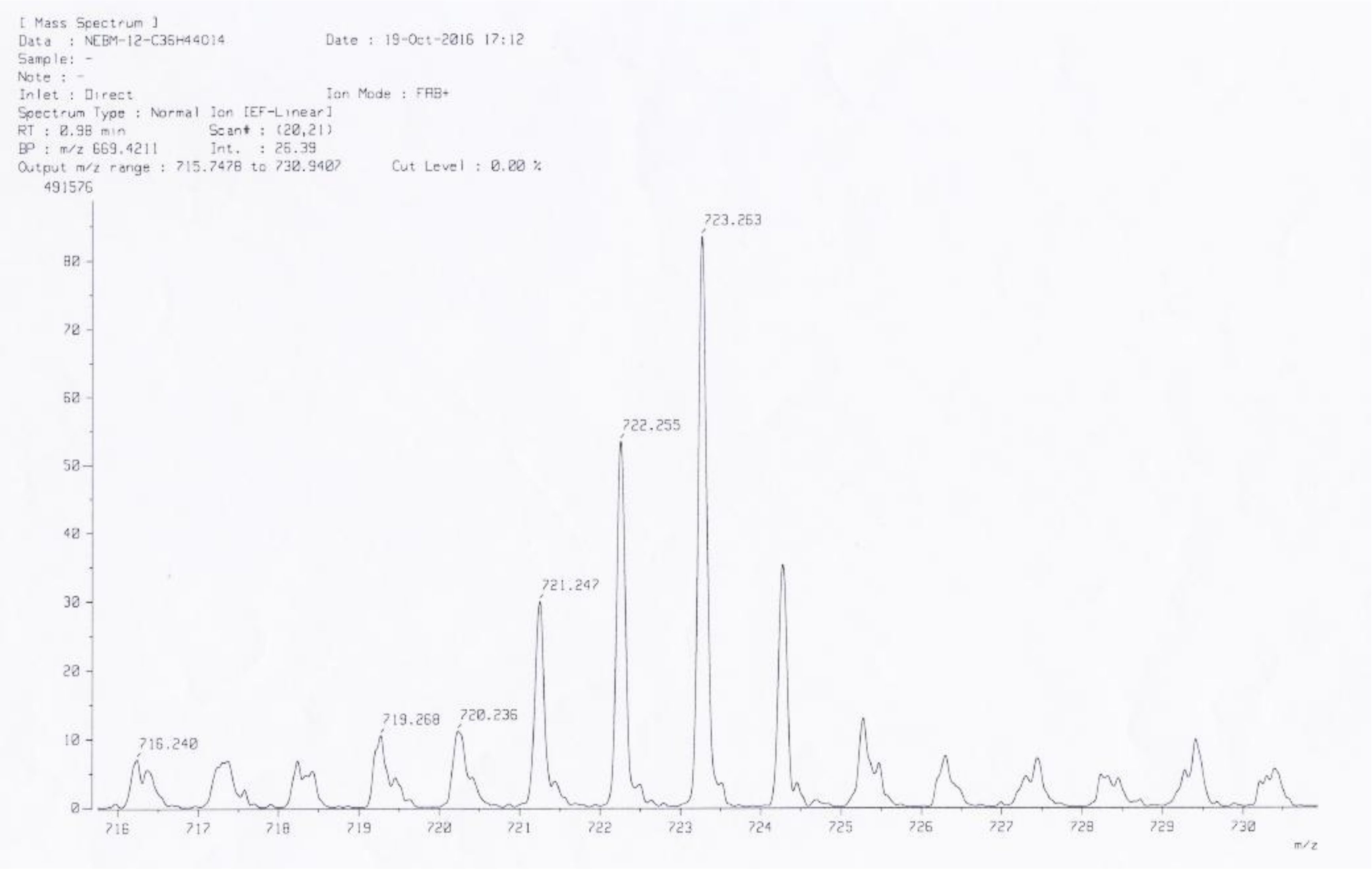

Fig. S7. HR-FAB-MS spectrum of **1**

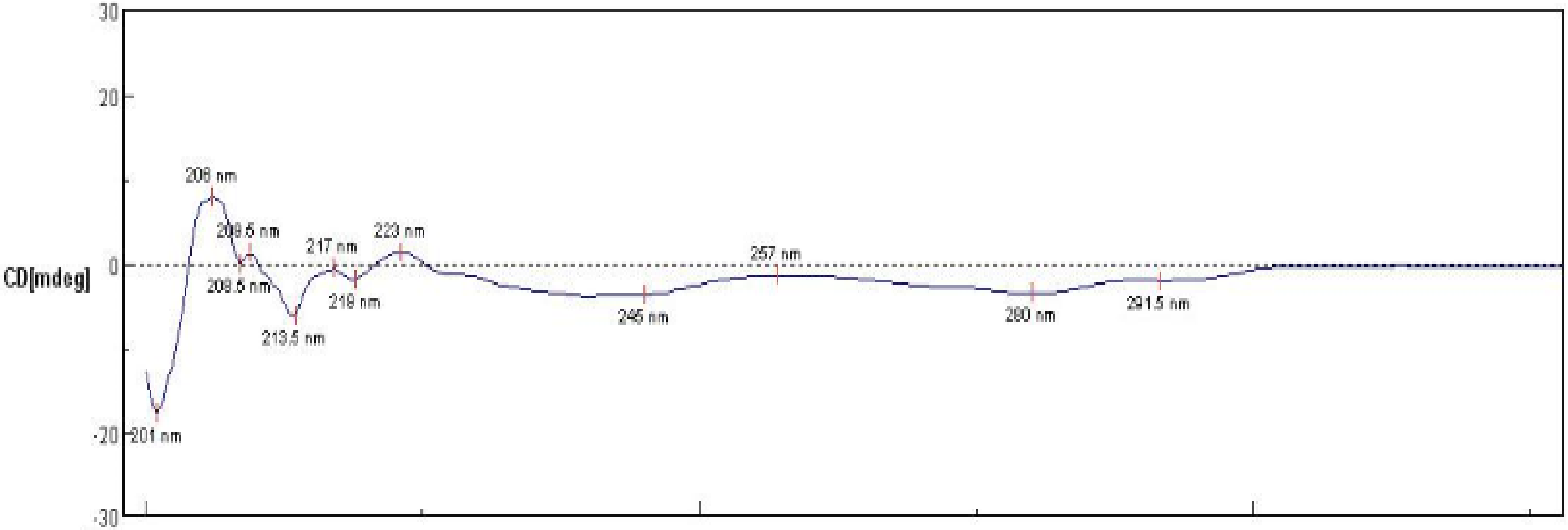

Fig. S8. CD spectra of compound **1**
